# Can Personality Traits Affect Sleep Quality in Post-COVID-19 Patients?

**DOI:** 10.3390/jcm14092911

**Published:** 2025-04-23

**Authors:** Anna Carnes-Vendrell, Gerard Piñol-Ripoll, Mar Ariza, Neus Cano, Barbara Segura, Carme Junque, Javier Béjar, Cristian Barrue, Nautilus Project Collaborative Group, Maite Garolera

**Affiliations:** 1 Cognitive Disorders Unit, Cognition and Behaviour Study Group, Hospital Universitari Santa Maria, 25198 Lleida, Spain; acarnes@gss.cat; 2 Clinical Research Group for Brain, Cognition and Behaviour, Consorci Sanitari de Terrassa, 08227 Terrassa, Spain; mariza@ub.edu (M.A.); ncanom@cst.cat (N.C.); mgarolera@cst.cat (M.G.); 3 Departament de Ciències Bàsiques, Universitat Internacional de Catalunya (UIC), 08195 Sant Cugat, Spain; 4 Medical Psychology Unit, Department of Medicine, Universitat de Barcelona, 08035 Barcelona, Spain; bsegura@ub.edu (B.S.); cjunque@ub.edu (C.J.); 5 Institute of Neurosciences, University of Barcelona, 08036 Barcelona, Spain; 6 Institut d’Investigacions Biomèdiques August Pi i Sunyer (IDIBAPS), 08036 Barcelona, Spain; 7 Centro de Investigación Biomédica en Red Sobre Enfermedades Neurodegenerativas (CIBERNED), 08028 Barcelona, Spain; 8 Faculty of Informatics of Barcelona (FIB), Polytechnic University of Catalonia, 08242 Barcelona, Spain; bejar@cs.upc.edu (J.B.); cbarrue@cs.upc.edu (C.B.); 9 Neuropsychology Unit, Consorci Sanitari de Terrassa, 08227 Terrassa, Spain

**Keywords:** Big Five Model, personality traits, post-COVID-19 condition, sleep quality

## Abstract

**Objectives**: In the present study, we aimed (i) to describe the personality traits of a cohort of post-COVID-19 condition (PCC) patients compared with a healthy control (HC) group, (ii) to evaluate the relationship between sleep quality and personality traits, and (iii) to investigate whether this relationship differs according to disease severity. **Methods**: We included 599 participants from the Nautilus Project (ClincalTrials.gov IDs: NCT05307549 and NCT05307575) with an age range from 20 to 65 years old. Of 599 participants, 280 were nonhospitalized (mild PCC), 87 were hospitalized (hospitalized PCC), 98 were in the PCC-ICU, and 134 were in the HC group. We assessed sleep quality with the Pittsburgh Sleep Quality Index (PSQI) and personality traits with the NEO Five-Factor Inventory (NEO FFI). **Results**: We found that mild-PCC patients had higher scores of neuroticism than HCs (*p* < 0.001) and ICU-PCC patients did (*p* = 0.020). The higher the neuroticism score was, the higher the total PSQI score (B 0.162; *p* < 0.001), the worse the sleep latency (B 0.049; *p* < 0.001), the greater the degree of sleep disturbance (B 0.060; *p* < 0.001), the greater the use of sleeping medication (B 0.035; *p* = 0.033), and the greater the incidence of daytime disturbances (B 0.065; *p* < 0.001) among the PCC patients. High neuroticism is also an indicator of worse sleep quality in mild-PCC (t = 3.269; *p* 0.001) and hospitalized-PCC (t = 6.401; *p* < 0.001) patients and HCs (t = 4.876; *p* < 0.001) but not in ICU-PCC patients. **Conclusions**: Although neuroticism affected sleep quality in both the PCC patients and HCs, the clinical implications and magnitude of the relationship were more significant in the PCC group. Specific and multidimensional interventions are needed to treat sleep problems in this population, and the influence of their personality traits should be considered.

## 1. Introduction

Sleep disturbances are among the most prevalent symptoms in post-COVID-19 condition (PCC) patients, along with cognitive [1,2,3,4,5,6,7] and emotional alterations [8,9]. Poor sleep quality is a type of sleep disturbance that has been widely studied, showing that there is a reduction in sleep quality in both hospitalized and nonhospitalized-PCC patients [10,11,12,13,14]. However, in a previous study, we found no significant correlation between sleep quality and the severity of the disease in PCC patients [15].

Personality traits related to the COVID-19 pandemic have been studied since its beginning. These are stable characteristics that reveal patterns of behaviour, habits, feelings and thoughts. The Big Five Model (Costa and McCrae 1990) is one of the tools most frequently used to characterize personality [16]. Five dimensions are represented in this model: extraversion, agreeableness, conscientiousness, neuroticism, and openness. To date, most studies have focused on analysing the associations between personality traits and the sequelae of the COVID-19 pandemic. A recent systematic literature review on how personality traits can influence the response to the COVID-19 pandemic reported that personality traits were correlated with the effects of COVID-19 and concluded that there was a positive relationship between neuroticism and anxiety during the COVID-19 pandemic [16]. Additionally, the associations among anxiety, depression, posttraumatic stress disorder, and premorbid personality traits have been studied [17,18].

However, to our knowledge, only two previous studies have focused on the role of personality traits in PCC patients. The first study examined the correlation between the neuropsychiatric features of post-COVID-19 syndrome and the main personality traits [19]. The second explored the potential link between personality profiles, specifically type D personalities, and an increased risk of long-term COVID-19 [20].

It is well known that sleep plays a fundamental role in the regulation of emotions and adequate cognitive functioning. In addition, there is evidence that five-factor model personality traits are associated with sleep. Sutin et al. reported in a population-based study that high neuroticism and low extraversion and conscientiousness were associated with more frequent wakefulness after sleep onset, greater fragmentation, and feelings of being less rested [21]. Another study with healthy undergraduate students revealed that conscientiousness and extraversion were the key personality predictors of sleep outcomes, whereas neuroticism, agreeableness, and openness to experience were not significantly related to sleep [22].

The lack of studies in this field in PCC patients, in addition to the established role of personality traits in sleep, has led us to study the relationship between personality traits and sleep in PCC patients. Therefore, in the present study, we aimed (i) to describe the personality traits of a cohort of PCC patients compared with a healthy control group, (ii) to evaluate the relationship between sleep quality and personality traits, and (iii) to investigate whether this relationship differs according to disease severity.

## 2. Materials and Methods

### 2.1. Participants and Study Design

We included 599 participants from the Nautilus Project (ClincalTrials.gov IDs: NCT05307549 and NCT05307575), of whom 465 had PCC and 134 were healthy controls (HCs). Among the PCC patients, during the acute phase of COVID-19, 280 were nonhospitalized (mild PCC), which means that only mild COVID-19 symptoms were observed; 87 were hospitalized; and 98 were admitted to the ICU. These latter two groups had severe complications, such as pneumonia, that needed hospitalization. This was a cross-sectional study, and the sample was recruited across 16 hospitals in Spain and Andorra consecutively. It was coordinated by the Consorci Sanitari de Terrassa (Terrassa, Barcelona, Spain). Recruitment was carried out between June 2021 and October 2022.

The inclusion criteria for the PCC group were a confirmed diagnosis of COVID-19 according to the WHO criteria with signs and symptoms of the disease observed during the acute phase, a period of at least 12 weeks after infection, and age between 18 and 65 years. The exclusion criteria were an established diagnosis of a psychiatric disorder, neurological disorder, neurodevelopmental disorder, or systemic pathology known to cause cognitive deficits before COVID-19 infection and motor or sensory alterations that could interfere with the neuropsychological assessment. The HCs had not had COVID-19 infection (no positive tests or compatible symptoms), and the same exclusion criteria for the PCC group were applied to the HC group.

### 2.2. Procedure and Instruments

The recruitment and data collection procedures have been previously described in another study [15]. Potential participants who referred persistent symptoms were referred mainly by neurologists, internists, or general practitioners to the investigators of each centre. Participation was completely voluntary, and we obtained written informed consent from all the participants before inclusion. No financial reward was provided to the participants, only information about the results of the evaluations we conducted. We collected data on sociodemographic characteristics, previous comorbidities, and COVID-19 symptoms in the first session. This information was collected in order to obtain full information about participants and their disease experience, as this was analysed in previous studies of our research group. Additionally, participants were given questionnaires to complete online or on paper to assess different variables. In this study, we focused on sleep quality, which was assessed with the Pittsburgh Sleep Quality Index (PSQI) [23], and premorbid personality, which was assessed with the NEO Five-Factor Inventory (NEO FFI) [24].

– **Pittsburgh Sleep Quality Index**: Each component of the PSQI questionnaire ranges from 0 to 3, with the total sum ranging from 0 to 21 points. Higher scores indicate poorer sleep quality, with a score greater than 5 suggesting significant sleep difficulties. With the questionnaire, seven subscales are obtained: subjective sleep quality, sleep latency, sleep efficiency, sleep duration, sleep disturbances, sleep medication, and daytime dysfunction. It has good psychometric properties, with sensitivity of 89.6% and specificity of 86.6% [23]. The internal consistency and reliability coefficient (Cronbach’s alpha) of PSQI is 0.83 for its seven components [23].
– **NEO Five-Factor Inventory**: It contains 60 items, and each of them uses a five-point Likert response format. It measures five domains: neuroticism, extraversion, openness, agreeableness, and conscientiousness. Internal consistency reliability ranged from 0.86 to 0.92 in the original study by Costa and McCrae [24]. It also has good psychometric properties in a Spanish validation study, whereas Cronbach’s alpha ranged from 0.66 to 0.81 [25,26].

The participants’ anonymity and confidentiality were guaranteed. The Scientific Ethics Committee of the Hospital Universitari Arnau de Vilanova approved both the study and the consent procedure (CEIC 2384), as did the Drug Research Ethics Committee (CEIm) of Consorci Sanitari de Terrassa (CEIm code: 02-20-107-070) and the Ethics Committee of the University of Barcelona (IRB00003099). Additionally, the investigation was conducted in accordance with the latest version of the Declaration of Helsinki.

### 2.3. Statistical Analysis

Descriptive analyses were performed on the HCs and PCC patients (mild, hospitalized, and ICU). For categorical variables, frequencies and percentages were registered, and for quantitative variables, the means and standard deviations were obtained. For both the sociodemographic and clinical profiles and the personality questionnaire, continuous parameters were compared between severity groups via nonparametric Kruskal–Wallis tests given the nonnormality of the data (checked via the Shapiro–Wilk test). For categorical parameters, groups were compared using Pearson’s chi2 test (with Fisher’s exact test, if appropriate). All multiple comparisons were adjusted by the Bonferroni correction.

To analyse the relationships between the total PSQI score and personality traits, multiple linear regression models were applied. To analyse the relationships between the different subscales of the PSQI (measured as ordinal variables), ordinal regression models were applied. These models were adjusted for sex and age. These multivariate models were also applied to 5 groups of participants: all PCC patients, mild-PCC patients, hospitalized-PCC patients, ICU-PCC patients and HCs. All the models were run with powerful estimation (robust covariances) to handle possible violations of model assumptions such as normality of distributions.

Robust covariances are an adjustment made to the variance and covariance estimates in statistical models, such as mixed models, to make them more reliable when certain model assumptions are not fully met. These assumptions, such as normality, homoskedasticity, or independence of errors, may fail in real-life cases. Robust covariances adjust the variance–covariance matrix of the estimated coefficients to make the model insensitive to heteroskedasticity and measurement errors, thereby obtaining valid results when classical assumptions are not met and improving the ability to make accurate inferences.

The statistical significance level that was used in the analyses was 5% (α = 0.05). All of the analyses were performed with IBM SPSS statistics 26.

## 3. Results

### 3.1. Description of the Sample

Among the 465 PCC patients, 280 had mild PCC (mean age 48.32 years, standard deviation (SD) 9.44), 87 were hospitalized (53.91, SD 8.80), and 98 were admitted to the ICU (53.25, SD 8.23). In the mild-PCC group, most patients were female (79.64%), whereas in the hospitalized group, the majority were male (51.14%). Participants in the ICU-PCC group consumed more alcohol (39.80%), were more obese (53.06%), and had more previous comorbidities, such as high blood pressure (29.59%) and dyslipidaemia (21.43%). Table 1 shows all the sociodemographic and clinical characteristics of the sample.

With respect to sleep quality, the mild-PCC patients had a mean PSQI total score of 9.54 (SD: 4.15), the hospitalized-PCC patients had a mean score of 7.82 (SD: 4.46), and the ICU patients had a mean score of 8.46 (SD: 4.38). When the percentage of patients who obtained ≥5 points on the PSQI, which indicates poor quality of sleep, was analysed, we found significant differences among the groups (*p* < 0.001). The healthy control group had a lower percentage of responses above 5 on the PSQI (41.60%) than did the mild-PCC (80.22%) group and hospitalized-PCC (61.90%) and ICU-PCC (67.39%) groups (Table 1).

### 3.2. Description of Personality Traits According to PCC Severity

The results of the personality trait assessment with the NEO-FFI questionnaire are presented in Table 2. We only found significant differences (*p* < 0.001) in the neuroticism subscale between the PCC participants and HCs. When the post hoc analysis was performed, we found that mild-PCC patients obtained higher scores than HCs did (post hoc contrast *p* < 0.001) and that mild-PCC patients had higher scores than ICU-PCC patients did (post hoc contrast *p* = 0.020).

### 3.3. Sleep Quality Results According to Personality Traits

When we analysed sleep quality according to personality traits in the PCC participants, we found some significant results, specifically with respect to neuroticism (see Table 3). The greater the degree of neuroticism is, the greater the total PSQI score (B 0.162; *p* < 0.001), which indicates poorer sleep quality. Conversely, the higher the extraversion score is, the lower the total PSQI score (B = −0.085; *p* = 0.019), which indicates better sleep quality. With respect to the subscales of the PSQI, we found that when neuroticism increases, the probability of having poorer subjective sleep quality (B 0.065; *p* < 0.001), worse sleep latency (B 0.049; *p* < 0.001), more sleep disturbances (B 0.060; *p* < 0.001), and the use of sleeping medication (B 0.035; *p* = 0.033), as well as daytime disturbances, increases (B 0.065; *p* < 0.001). Furthermore, as openness decreases (B = −0.071; *p* = 0.030) and conscientiousness increases (B 0.065; *p* = 0.002), we also observe that sleep efficiency worsens. Finally, as conscientiousness increases (B 0.042; *p* = 0.040), sleep duration decreases.

Finally, we also analysed the influence of personality traits on sleep quality in terms of the severity of the PCC and in the healthy control group (see Table 4). In summary, higher neuroticism is also an indicator of worse sleep quality (both in the total PSQI score and the subjective sleep quality subscale) in mild-PCC and hospitalized-PCC patients, and it also indicates longer sleep latency, shorter sleep duration, sleep disturbances, and daytime dysfunction in the same groups. However, neuroticism did not affect the sleep quality of the ICU-PCC patients. In this group, we found that more conscientiousness indicates more sleep efficiency, and that less extraversion indicates more daytime dysfunction. Similar results were found in the HC group, with neuroticism being the main personality trait that correlated with worse sleep quality.

## 4. Discussion

Our study demonstrated the effects of personality traits, especially neuroticism, on the sleep quality of PCC participants. In fact, when we compared the results of the NEO-FII questionnaire among groups of participants stratified by disease severity, we found that mild-PCC patients had more traits of neuroticism than HCs and ICU-PCC patients did. Regarding the second and third aims of this study, we found that neuroticism is the personality trait that is most strongly associated with sleep quality in the PCC and HC groups.

One previous study reported the same pattern of neuroticism in PCC participants. The authors reported that there were significant differences in emotional stability (which indicates inverse scores of neuroticism with the BFSI) between post-COVID-19 syndrome patients and healthy controls [19]. However, their sample of PCC participants was smaller than ours, and they did not analyse disease severity.

In addition to the descriptive results, we found that PCC participants with higher levels of neuroticism had poorer sleep quality. This result was confirmed when PCC participants with higher traits of extraversion reported better sleep quality. In the same previous study mentioned above, the authors did not find significant correlations between any of the five personality factors and sleep quality [19]. However, in a population-based study with older adult participants, neuroticism was associated with feeling less rested, whereas higher extraversion and conscientiousness were associated with feeling more rested [21]. Although the authors did not use a standardized questionnaire to assess sleep quality in that study, they did obtain objective measures with actigraphy. The conclusion of that study was that neuroticism was associated with more waking after sleep onset and greater fragmentation [21]. In our study, we also found that greater neuroticism was related to worse sleep latency, more sleep disturbances, more use of sleeping medications, and more daytime disturbances. Although this study is based on the general population, we can deduce that PCC participants obtain results similar to those of the general population regarding the effect of neuroticism on sleep quality.

Amsterdam et al. [20] studied the link between distinctive personality profiles, specifically type D personalities, and an increased risk of long-term COVID-19. The type distressed personality (D) is a concept used in psychology to define a tendency towards negative affectivity and social inhibition. Individuals with a type D personality tend to experience increased negative emotions across time and situations and tend not to share these emotions with others because of fear of rejection or disapproval. The negative affectivity characteristic of the type D personality reflects the tendency to experience negative emotions, including depressed mood, worry, anxiety, helplessness, and sadness. Therefore, in psychology, it overlaps with many aspects of the neuroticism trait. As type D personality has been linked to several medical conditions, such as ischaemic heart disease [27,28], Amsterdam et al. wanted to determine whether long-term COVID-19 patients are more likely to have this type of personality. They performed a cluster analysis within the participants, and Cluster 1 was formed by long-COVID-19 patients with type D personalities meeting the criteria. Furthermore, they compared participants from this group with other long-COVID-19 patients with no criteria for type D personality with respect to different variables, including sleep quality. They reported that the same group of patients with long-term COVID-19 and type D personality had significantly poorer sleep quality [20].

We wanted to go a step further and analyse the same relationship between personality traits and sleep quality but with regard to the severity of the disease. We found that greater neuroticism was also an indicator of worse sleep quality in mild-PCC and hospitalized-PCC patients and indicated longer sleep latency, sleep duration, sleep disturbances, and daytime dysfunction in the same groups. However, and unexpectedly, neuroticism did not correlate with the sleep quality of the ICU-PCC patients. According to our previous study, anxiety levels predict poor sleep quality in the ICU-PCC group [15]. If we search the literature, anxiety has always been linked to higher levels of neuroticism [18]. Therefore, anxiety is a better predictor of sleep quality than neuroticism is in ICU-PCC patients.

Finally, we cannot forget that in the HC group, neuroticism also explains poor sleep quality, but its severity and the variables affected are greater in PCC patients due to interactions with other factors of the disease. We must not forget that neuroticism is a personality trait that negatively impacts sleep quality in any population [21,22], including the general population, as mentioned above. However, compared with HCs, PCC patients are exposed to additional factors (physiological, psychological, and emotional) that amplify this relationship, which causes sleep quality to worsen in PCC participants compared with HCs. Although neuroticism affects sleep quality in both the PCC and HC groups, the cumulative effects in PCC patients exacerbate this relationship. It should also be noted that specific patterns of sleep disturbance may differ: in HCs, the relationship between neuroticism and sleep could be reflected mainly in subjective perceptions (poor subjective sleep quality) or mild disturbances, whereas in PCC participants, the relationship also manifested as a greater and more objective impact, such as greater sleep disturbances, the use of medication, or daytime dysfunction. Furthermore, we must not forget that our HC is a control group that experienced the effects of the pandemic and therefore was not free from stressors related to COVID-19. Factors such as uncertainty and social restrictions experienced during the pandemic can interact with neuroticism and thus affect the quality of sleep in HC participants, as has been observed in other studies [29,30,31,32]. Furthermore, a previous study highlighted the critical role of sleep quality in managing stress-induced somatic symptoms resulting from the pandemic in a study conducted with teachers [33]. 

### Future Research and Limitations

Our findings have potential implications for treatment. If we can identify people with specific personality traits, we can predict those who are likely to have greater impairments in sleep quality, which means that we can implement preventative therapeutic strategies. On the other hand, based on the relationship between sleep quality and some personality traits, treatments focused on improving sleep quality may result in higher levels of extraversion and less neuroticism. In the same way, psychological approaches such as psychotherapy for reducing neuroticism levels may also have a positive effect in the sleep quality.

However, when interpreting the results, some limitations must be considered. We did not collect information about previous sleep disturbances prior to COVID-19 infection. Also, some potential confounding factors such as socioeconomic status or psychological impact of the COVID-19 pandemic may have influenced both personality traits and sleep quality results. Additionally, we have the limitations inherent to a cross-sectional and correlational study, such as the impossibility of making causal predictions (cause–effect) and selection bias (as it is a study with consecutive recruitment). Instead, we collected several pieces of information from a large sample of PCC participants, including a healthy control group, which allowed us to perform robust statistical analysis to determine the relationships between sleep quality and personality traits, and all the analyses were adjusted for age and sex.

## 5. Conclusions

Although the COVID-19 pandemic has ended, the sequelae of COVID-19 are still present in the population, and the disease may have a long-lasting impact on health due to persistent COVID-19 symptoms. We did find significant differences in personality traits, specifically neuroticism levels, among the PCC patients by disease severity. Furthermore, our results indicated that high levels of neuroticism affect sleep quality. These results have been previously described among the general population, so our study confirms that PCC patients experience the same relationships between personality traits and subjective sleep quality. Although this relationship was observed in both the PCC and HC groups, the magnitude of the relationship was more significant in the PCC group; thus, the clinical implications are also more relevant. Therefore, these results have implications for PCC patients, who are more likely to experience sleep disturbances according to their personality traits and who may require more specific and multidimensional interventions.

## Figures and Tables

**Table 1:** Clinical and sociodemographic characteristics of the sample.

	Healthy Controls	Mild PCC	Hospitalized PCC	ICU PCC	*p* Value
	N = 134	N = 280	N = 87	N = 98	
Age (years) (SD)	47.36 (10.13)	48.32 (9.44)	53.91 (8.80)	53.25 (8.23)	<0.001 ***
Female (%)	74.63%	79.64%	48.86%	50.00%	<0.001 ***
Years of education (SD)	15.63 (3.00)	14.48 (3.18)	13.27 (3.47)	13.22 (3.29)	<0.001 ***
MoCA (SD)	27.87 (1.79)	26.29 (2.72)	25.63 (3.01)	25.31 (2.87)	<0.001 ***
BMI (SD)	25.32 (6.04)	25.75 (5.03)	27.90 (5.22)	31.07 (5.26)	<0.001 ***
Tobacco smoking (%)	24.63%	10.14%	5.68%	6.12%	<0.001 ***
Alcohol consumption (%)	28.36%	23.19%	28.41%	39.8%	0.019 *
**Civil status**					
Married (%)	50.75%	60.5%	60.23%	66.33%	0.1
**Previous** **comorbidities**					
Heart disease (%)	2.24%	3.62%	3.41%	4.08%	0.865
Respiratory disease (%)	4.48%	14.13%	15.91%	14.29%	0.019 *
Chronic kidney disease (%)	0.00%	0.72%	1.14%	2.04%	0.396
High blood pressure (%)	4.48%	9.42%	20.45%	29.59%	<0.001 ***
Dyslipidaemia (%)	11.19%	9.78%	18.18%	21.43%	<0.001 ***
Diabetes mellitus (%)	2.24%	1.09%	10.23%	9.18%	<0.001 ***
Obesity (%)	12.69%	18.84%	34.09%	53.06%	<0.001 ***
Chronic liver disease (%)	0.00%	1.45%	5.68%	4.08%	0.015 *
Chronic pain (%)	5.3%	5.73%	16.47%	7.22%	0.007 **
**Quality of sleep**					
PSQI total score (SD)	5.42 (3.25)	9.54 (4.15)	7.82 (4.46)	8.46 (4.38)	<0.001 ***
Poor quality of sleep (>5)	41.6%	80.22%	61.9%	67.39%	<0.001 ***

Unless otherwise specified, The results are presented as the means (standard deviations). Statistical significance = * *p* < 0.05. ** *p* < 0.01. *** *p* < 0.001. PCC: post-COVID-19 condition; MoCA: Montreal Cognitive Assessment; BMI: body mass index.

**Table 2:** Description of personality traits according to PCC severity.

NEO-FFI	Healthy Controls	Mild PCC	Hospitalized PCC	ICU PCC	*p* Value
Neuroticism	18.96 (8.19)	23.06 (8.08)	21.16 (8.68)	20.26 (8.73)	<0.001 ***
Extraversion	29.48 (7.07)	27.63 (6.9)	29.03 (7.27)	27.54 (6.88)	0.076
Openness	30.08 (4.13)	29.7 (4.39)	28.96 (4.35)	28.96 (4.51)	0.124
Agreeableness	30.99 (5.28)	30.61 (4.89)	30.43 (4.92)	30.56 (5.71)	0.682
Conscientiousness	31.87 (4.36)	31.37 (5.80)	31.33 (5.20)	32.15 (6.37)	0.713

The results are presented as the means (standard deviations). Statistical significance = *** *p* < 0.001. PCC: Post-COVID-19 condition.

**Table 3:** Regression analyses of sleep quality and personality traits of PCC participants.

**PSQI Total Score**	**Unstandardized** **Coefficients**		**t ^a^**	**Sig.**	**95.0% C.I. for B**	
	**B**	**Standard Deviation**			**Lower**	**Upper**
NEO-FFI: Neuroticism	0.162	0.0312	5.183	<0.001 ***	0.100	0.223
NEO-FFI: Extraversion	−0.085	0.0362	−2.349	0.019 *	−0.156	−0.014
NEO-FFI: Openness	−0.010	0.0612	−0.170	0.865	−0.131	0.110
NEO-FFI: Agreeableness	0.004	0.0454	0.081	0.935	−0.086	0.093
NEO-FFI: Conscientiousness	0.035	0.0394	0.889	0.375	−0.042	0.113
**PSQI Subscales**	**Unstandardized Coefficients**		**Wald ^b^**	**Sig.**	**95.0% C.I. for B**	
	**B**	**Standard Deviation**			**Lower**	**Upper**
**Subjective sleep quality**						
NEO-FFI: Neuroticism	0.065	0.015	18.445	<0.001 ***	0.036	0.095
NEO-FFI: Extraversion	−0.021	0.017	1.484	0.223	−0.054	0.013
NEO-FFI: Openness	0.012	0.030	0.170	0.680	−0.047	0.072
NEO-FFI: Agreeableness	−0.038	0.022	2.911	0.088	−0.082	0.006
NEO-FFI: Conscientiousness	0.021	0.019	1.223	0.269	−0.016	0.058
**Sleep latency**						
NEO-FFI: Neuroticism	0.049	0.015	10.876	0.001 **	0.020	0.079
NEO-FFI: Extraversion	−0.036	0.017	4.473	0.034	−0.069	−0.003
NEO-FFI: Openness	0.034	0.030	1.312	0.252	−0.024	0.093
NEO-FFI: Agreeableness	−0.001	0.022	0.001	0.971	−0.044	0.043
NEO-FFI: Conscientiousness	0.023	0.019	1.442	0.230	−0.014	0.060
**Sleep efficiency**						
NEO-FFI: Neuroticism	0.026	0.016	2.556	0.110	−0.006	0.059
NEO-FFI: Extraversion	−0.033	0.019	3.034	0.082	−0.069	0.004
NEO-FFI: Openness	−0.071	0.033	4.694	0.030 *	−0.136	−0.007
NEO-FFI: Agreeableness	0.047	0.025	3.603	0.058	−0.002	0.096
NEO-FFI: Conscientiousness	0.065	0.021	9.256	0.002 **	0.023	0.106
**Sleep duration**						
NEO-FFI: Neuroticism	0.057	0.016	12.647	0.000	0.026	0.088
NEO-FFI: Extraversion	0.008	0.018	0.188	0.664	−0.027	0.043
NEO-FFI: Openness	−0.043	0.032	1.798	0.180	−0.105	0.020
NEO-FFI: Agreeableness	−0.004	0.024	0.026	0.873	−0.050	0.042
NEO-FFI: Conscientiousness	0.042	0.020	4.236	0.040 *	0.002	0.081
**Sleep disturbances**						
NEO-FFI: Neuroticism	0.060	0.016	13.528	<0.001 ***	0.028	0.092
NEO-FFI: Extraversion	−0.035	0.018	3.548	0.060	−0.071	0.001
NEO-FFI: Openness	0.031	0.032	0.908	0.341	−0.032	0.094
NEO-FFI: Agreeableness	0.026	0.024	1.193	0.275	−0.021	0.073
NEO-FFI: Conscientiousness	−0.010	0.020	0.256	0.613	−0.050	0.030
**Sleep medication**						
NEO-FFI: Neuroticism	0.035	0.016	4.543	0.033 *	0.003	0.067
NEO-FFI: Extraversion	−0.030	0.019	2.562	0.109	−0.066	0.007
NEO-FFI: Openness	−0.018	0.033	0.295	0.587	−0.081	0.046
NEO-FFI: Agreeableness	0.018	0.024	0.569	0.451	−0.029	0.066
NEO-FFI: Conscientiousness	−0.034	0.021	2.678	0.102	−0.074	0.007
**Daytime dysfunction**						
NEO-FFI: Neuroticism	0.065	0.017	15.545	<0.001 ***	0.033	0.097
NEO-FFI: Extraversion	−0.032	0.019	2.893	0.089	−0.068	0.005
NEO-FFI: Openness	0.013	0.033	0.145	0.703	−0.052	0.077
NEO-FFI: Agreeableness	−0.028	0.024	1.291	0.256	−0.075	0.020
NEO-FFI: Conscientiousness	−0.017	0.021	0.709	0.400	−0.058	0.023

Statistical significance = * *p* < 0.05, ** *p* < 0.01, *** *p* < 0.001. ^a^ t statistic derived from the null test of the beta coefficient of the multiple linear regression between the total PSQI score and the personality traits. ^b^ Wald statistic derived from the null test of the beta coefficient of the ordinal regression between the PSQI parameters and the personality traits.

**Table 4:** Regression analyses of sleep quality and personality traits for all subgroups.

	Mild PCC	Hospitalized PCC	ICU PCC	Healthy Controls
**PSQI Total Score**				
NEO-FFI: Neuroticism	0.131 ** (t = 3.269; *p* = 0.001)	0.382 *** (t = 6.401; *p* < 0.001)		0.200 *** (t = 4.876; *p* < 0.001)
NEO-FFI: Extraversion				
NEO-FFI: Openness		−0.323 ** (t = -2.906; *p* = 0.005)		
NEO-FFI: Agreeableness				
NEO-FFI: Conscientiousness		0.243 * (t = 2.518; *p* = 0.014)		
**PSQI: Subjective Sleep Quality**				
NEO-FFI: Neuroticism	0.045 * (Wald(1) = 5.929; *p* = 0.024)	0.221 *** (Wald(1) = 23.335; *p* = 0.001)		0.079 *** (Wald(1) = 12.339; *p* < 0.001)
NEO-FFI: Extraversion				
NEO-FFI: Openness		−0.179 * (Wald(1) = 5.339; *p* = 0.021)		
NEO-FFI: Agreeableness	−0.071 (Wald(1) = 5.217; *p* = 0.022)			
NEO-FFI: Conscientiousness		0.179 ** (Wald(1) = 8.591; *p* = 0.003)		
**PSQI: Sleep Latency**				
NEO-FFI: Neuroticism	0.044 * (Wald(1) = 5.026; *p* = 0.025)	0.107 ** (Wald(1) = 7.502; *p* = 0.006)		0.068 ** (Wald(1) = 9.763; *p* = 0.002)
NEO-FFI: Extraversion				
NEO-FFI: Openness				
NEO-FFI: Agreeableness				
NEO-FFI: Conscientiousness				
**PSQI: Sleep Efficiency**				
NEO-FFI: Neuroticism				
NEO-FFI: Extraversion				
NEO-FFI: Openness		−0.185 * (Wald(1) = 4.840; *p* = 0.028)		
NEO-FFI: Agreeableness				
NEO-FFI: Conscientiousness			0.091 * (Wald(1) = 3.925; *p* = 0.048)	
**PSQI: Sleep Duration**				
NEO-FFI: Neuroticism	0.050 * (Wald(1) = 5.744; *p* = 0.017)	0.164 *** (Wald(1) = 10.816; *p* < 0.001)		0.048 * (Wald(1) = 4.093; *p* = 0.043)
NEO-FFI: Extraversion				
NEO-FFI: Openness				
NEO-FFI: Agreeableness				
NEO-FFI: Conscientiousness				
**PSQI: Sleep Disturbance**				
NEO-FFI: Neuroticism	0.050 * (Wald(1) = 5.275; *p* = 0.022)	0.170 *** (Wald(1) = 14.234; *p* < 0.001)		0.072 ** (Wald(1) = 9.430; *p* = 0.002)
NEO-FFI: Extraversion				
NEO-FFI: Openness				
NEO-FFI: Agreeableness		0.126 * (Wald(1) = 4.431; *p* = 0.035)		
NEO-FFI: Conscientiousness				
**PSQI: Sleep Medication**				
NEO-FFI: Neuroticism		0.193 *** (Wald(1) = 12.509; *p* < 0.001)		0.107 *** (Wald(1) = 15.650; *p* < 0.001)
NEO-FFI: Extraversion				
NEO-FFI: Openness		−0.270 ** (Wald(1) = 8.239; *p* = 0.004)		
NEO-FFI: Agreeableness				
NEO-FFI: Conscientiousness	−0.053 * (Wald(1) = 4.001; *p* = 0.045)			
**PSQI: Daytime Dysfunction**				
NEO-FFI: Neuroticism	0.067 ** (Wald(1) = 9.144; *p* 0.002)	0.086 * (Wald(1) = 4.078; *p* 0.043)		0.063 ** (Wald(1) = 7.496; *p* 0.006)
NEO-FFI: Extraversion			−0.097 * (Wald(1) = 5.285; *p* 0.022)	
NEO-FFI: Openness				0.099 * (Wald(1) = 3.922; *p* 0.048)
NEO-FFI: Agreeableness				
NEO-FFI: Conscientiousness				

Statistical significance = * *p* < 0.05. ** *p* < 0.01. *** *p* < 0.001. Beta coefficient. Statistics (*p* value). Only *p* values less than 0.05 were used. Only *p* values less than 0.05 from the null test of the beta coefficient of the multiple linear regression or the ordinal regression relating the total PSQI score and the personality traits are shown. For the multiple linear regression (PSQI total score), the value of the t statistic is shown (t = value). For the ordinal regressions (PSQI subscales), the values of the Wald statistic and the degrees of freedom are shown (Wald(df) = value).

## Data Availability

The raw data supporting the conclusions of this article will be made available by the authors without undue reservation.
